# Evaluation of a State-Level Incentive Program to Improve Diet

**DOI:** 10.1001/jamanetworkopen.2025.44215

**Published:** 2025-11-18

**Authors:** Alison Tovar, Lisa M. Powell, Julien Leider, Emily Elenio, Vanessa M. Oddo, Maya K. Vadiveloo

**Affiliations:** 1 Department of Behavioral and Social Sciences, School of Public Health, Brown University, Providence, Rhode Island; 2 Division of Health Policy and Administration, School of Public Health, University of Illinois Chicago; 3 Institute for Health Research and Policy, School of Public Health, University of Illinois Chicago; 4 Department of Kinesiology and Nutrition, College of Applied Health Sciences, University of Illinois Chicago; 5 Department of Nutrition, College of Health Sciences, The University of Rhode Island, Kingston

## Abstract

**Importance:**

Fruit and vegetable incentive programs show promise for improving dietary quality among Supplemental Nutrition Assistance Program (SNAP) participants, but few programs have been implemented at scale. Understanding state-level program effectiveness is critical for informing nutrition assistance policy.

**Objective:**

To evaluate the association of Eat Well, Be Well (EWBW), the first state-level SNAP fruit and vegetable incentive program launched in Rhode Island, with dietary intake and program awareness during initial implementation.

**Design, Setting, and Participants:**

In this pre-post cohort study with a comparison site (Connecticut) using difference-in-differences analysis, baseline data were collected from May 15 to September 30, 2023, with follow-up from June 12 to October 6, 2024 (5-8 months after implementation). Community-based recruitment took place across Rhode Island (intervention) and Connecticut (comparison), including food pantries and health fairs, as well as via text messages sent to participants from the Special Supplemental Nutrition Program for Women, Infants, and Children. Participants were current SNAP recipients, aged 18 years or older, English or Spanish speaking and had email or text access.

**Intervention:**

Automatic enrollment providing a $0.50 credit per $1 spent on fresh fruits and vegetables (up to $25/mo) at 2 grocery chains, with credits deposited on Electronic Benefits Transfer cards.

**Main Outcomes and Measures:**

The primary outcome was change in total fruit and vegetable intake (measured as cup equivalents per 1000 kcal) via validated food frequency questionnaire. Secondary outcomes included diet quality (measured by the Healthy Eating Index–2015), program awareness, and use of the program.

**Results:**

Of the 725 participants (mean [SD] baseline age, 34.5 [10.1] years; 364 in Rhode Island and 361 in Connecticut), 690 were women (95.2%), and the mean (SD) household size was 3.9 (1.6) at baseline and follow-up. Baseline mean (SD) fruit and vegetable intake was 2.05 (1.19) cup equivalents per 1000 kcal in Rhode Island and 2.12 (1.25) cup equivalents per 1000 kcal in Connecticut. There was no significant change in fruit and vegetable intake in Rhode Island relative to Connecticut (0.12 [95% CI, −0.04 to 0.28] cup equivalents per 1000 kcal). Stratified analyses among those with higher baseline consumption of fruits and vegetables (>1.79 cup equivalents/1000 kcal) showed that fruit and vegetable intake significantly increased in Rhode Island relative to Connecticut (0.29 [95% CI, 0.03-0.54] cup equivalents per 1000 kcal), while those with lower baseline consumption of fruits and vegetables showed no change (0.00 [95% CI, −0.17 to 0.18] cup equivalents per 1000 kcal). A total of 134 of 364 Rhode Island respondents (36.8%) correctly identified the program’s purpose and 96 of 364 (26.4%) reported using discounts available through the EWBW.

**Conclusions and Relevance:**

In this cohort study of SNAP participants, the 50% incentive, automatic enrollment EWBW program was not associated with significant relative changes in fruit and vegetable intake, but was associated with benefits among participants already consuming more fruits and vegetables. Enhanced implementation, including broader retail partnerships, improved outreach, and targeted interventions for populations with lower intake of fruits and vegetables, may be needed to maximize public health outcomes.

## Key Points

**Question:**

Is a state-level Supplemental Nutrition Assistance Program (SNAP) fruit and vegetable incentive program with automatic enrollment associated with improved dietary intake among adults with low income?

**Findings:**

In this pre-post cohort study with a comparison site including 725 SNAP participants, the 50% incentive program resulted in a modest, nonsignificant increase in fruit and vegetable intake (0.12 cup equivalents per 1000 kcal). Participants with higher baseline consumption of fruits and vegetables showed significant increases (0.29 cup equivalents per 1000 kcal), while consumers with a lower baseline level showed no change.

**Meaning:**

This study suggests that financial incentives may be associated with improved diet among individuals already inclined toward healthy eating, indicating that targeted implementation strategies are needed.

## Teaser

This pre-post cohort study evaluates the association of a state-level Supplemental Nutrition Assistance Program fruit and vegetable incentive program with improved dietary intake and program awareness during initial implementation.

## Introduction

Although the Supplemental Nutrition Assistance Program (SNAP) was designed to reduce food insecurity and improve nutrition, significant disparities in diet quality persist between SNAP participants and nonparticipants, especially in comparison with adults with higher incomes.^1,2^ Fruit and vegetable incentives reduce financial barriers and improve dietary intake,^3,4,5^ although most programs have limited geographic reach.^6^

Although there is evidence that fruit and vegetable incentive programs reduce household food insecurity and increase fruit and vegetable consumption among populations with low income, there have been few attempts to scale these programs.^3^ A 2025 review found consistent positive outcomes across initiatives and highlighted the need for evaluation of long-term effects.^7^ However, implementation challenges remain, including restrictive eligibility criteria and access barriers for different populations. To date, programs such as the US Department of Agriculture’s Healthy Incentives Pilot (HIP) and Food Insecurity Nutrition Incentives (FINI) have used diverse incentive structures, including 30% rebates and dollar-for-dollar matches, with considerable success.^8,9^ In HIP, participants consumed 26% more targeted fruits and vegetables and spent 11% more SNAP benefits on targeted fruits and vegetables^10^; FINI projects showed 12% to 16% increases in fruit and vegetable purchases.^11^ Its successor, the Gus Schumacher Nutrition Incentive Program (GusNIP), has reported over $40 million in redeemed incentives alongside participant-reported increases in fruit and vegetable intake while generating approximately $100 million in economic impact.^12^ Beyond benefiting individuals with low income,^3^ fruit and vegetable incentives boost retailer sales^9,11^ and may reduce chronic disease costs.^12^ More research is needed on sustained dietary changes and factors for scalable implementation.^7,13^

Despite promising pilot evidence, gaps remain in understanding the effectiveness and scalability of state-level fruit and vegetable incentives. Behavioral economics further underscores the importance of evaluating program design: features such as automatic enrollment and seamless integration across retailers may serve as structural nudges that amplify outcomes.^14,15^ Although fruit and vegetable incentive programs improve food security and intake, empirical assessments of effectiveness beyond use of the program remain limited.^7^ The Rhode Island Eat Well, Be Well (EWBW) program offers a unique opportunity to address this gap by assessing changes in fruit and vegetable intake after the launch of the first state-level incentive delivered via Electronic Benefits Transfer (EBT). The program automatically enrolled all households receiving SNAP benefits and provided a credit on any fresh fruit and vegetable purchase at participating retailers.

This study examines the short-term associations of EWBW with SNAP participants’ dietary outcomes and contributes to the growing body of evidence on nutrition incentives.^14^ We hypothesized that, compared with SNAP participants in the comparison site (Connecticut), those in the intervention site (Rhode Island) would demonstrate greater improvements in fruit and vegetable intake (primary outcome) 5 to 8 months after implementation of the EWBW program.

## Methods

The EWBW program launched in January 2024, automatically delivering a fruit and vegetable incentive to all SNAP households via EBT cards, without the need to take any action or receive formal notification. The EWBW program provides a $0.50 credit for every $1 spent on fresh fruits and vegetables (excluding canned or frozen fruits and vegetables or legumes) up to $25 per month at eligible retailers: Stop & Shop and Walmart. Stop & Shop represents 43% of the market share in Rhode Island and 40% in Connecticut.^16^ An initial marketing campaign was launched to publicize the program at rollout. The What’s On Your Plate study was developed to independently evaluate the EWBW program. The study is assessing both short-term (5-8 months) and longer-term (17-20 months after implementation) changes in fruit and vegetable intake. The study was approved by the institutional review boards at the University of Rhode Island and Brown University. All participants provided written informed consent. This study followed the Strengthening the Reporting of Observational Studies in Epidemiology (STROBE) reporting guideline for cohort studies.

### Population

The What’s On Your Plate study uses a convenience sample and a longitudinal pre-post study design, using Connecticut as a comparison site given its proximity and comparable sociodemographic profile to Rhode Island. SNAP participants were recruited before implementation (May 15-September 30, 2023). We determined that a target sample size of 1250 (625 per state) was needed to detect a 0.25-cup difference in fruit and vegetable intake between the sites, accounting for 20% attrition after 6 months.^17^

### Design and Flow

Eligible participants were 18 years of age or older, English or Spanish speaking, current SNAP recipients (self-reported), Rhode Island or Connecticut residents, with access to email, access to a text-capable phone, and provided consent. To recruit participants, the team collaborated with the Special Supplemental Nutrition Program for Women, Infants, and Children (WIC) by sending out text blasts, and with community partners, who distributed flyers and QR code cards.

Participants first completed a brief screening via Qualtrics to verify eligibility. Eligible participants then filled out a food frequency questionnaire (FFQ) through VioScreen^18^ and a sociodemographic survey on Qualtrics. Automated checks preceded $50 incentives; suspicious cases were reverified with follow-up contacts. The baseline participant list (n = 1363) was used to recontact participants. Between June 12 and October 6, 2024, participants verified their SNAP status by uploading grocery receipts showing the last 4 EBT digits. Duplicate EBT numbers from within the same state were contacted and asked to verify their information. All verified participants who responded to follow-up outreach (n = 900) were asked to complete a second FFQ and sociodemographic questionnaire.

**Figure 1.**
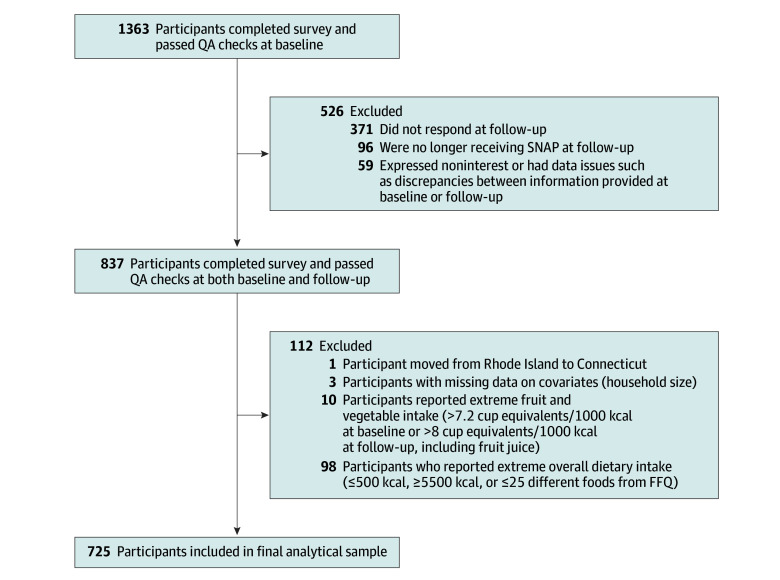
Participant Flow and Exclusion Criteria for Final Analytical Sample FFQ indicates food frequency questionnaire; QA, quality assurance; and SNAP, Supplemental Nutrition Assistance Program.

Among the 1363 participants who were recontacted, 371 (27.2%) did not respond. Of the 992 participants who responded to follow-up invitations, 96 (9.7%) were not eligible because they were no longer receiving SNAP benefits, and 59 (5.9%) were excluded due to noninterest, discrepancies between baseline and follow-up information, or other data issues, leaving 837 participants who successfully completed follow-up data collection. Last, we applied similar exclusion criteria to dietary data as in previous analyses of the baseline data, including extreme dietary intake,^17^ resulting in a final analytic sample of 725 participants (364 in Rhode Island and 361 in Connecticut) (Figure 1). Casewise deletion was used in limited cases of missing data.

### Survey Measures

Participants reported on personal and household characteristics (eg, marital status, housing, race and ethnicity [Hispanic, non-Hispanic Black or African American, non-Hispanic White, and non-Hispanic other race or ethnicity (American Indian or Alaska Native, Asian, Native Hawaiian, Other Pacific Islander, do not know or not sure, >1 race, or a self-described category that did not align with standard classifications)]), along with questions related to food shopping and consumption patterns and barriers and food and nutrition security. At follow-up, additional questions were added to assess participants’ awareness and understanding of the EWBW program, including multiple-choice items asking participants to describe the purpose of the program, the types of foods eligible for discounts, and whether they had personally used the discounts. We collected self-identified race and ethnicity to describe sample representativeness, to account for baseline compositional differences between the Rhode Island and Connecticut cohorts, and to be able to assess whether program reach and dietary impacts differed across groups. We treat race and ethnicity as social constructs that proxy exposure to structural inequities, not biological determinants.

Dietary intake was assessed using VioScreen’s validated 155-item, semiquantitative online 3-month FFQ that estimates usual intake and dietary quality, using the Healthy Eating Index–2015, which is interchangeable with the Healthy Eating Index–2020.^19^ VioScreen’s design, which includes graphics and branching logic, takes approximately 20 to 25 minutes to complete, minimizing participant burden.^18^

Fruit and vegetable intake was designated as the primary outcome, consistent with the intent of EWBW. Diet quality and program awareness were designated as secondary outcomes. Although EWBW targets fruit and vegetable consumption directly, improvements in overall diet quality may occur indirectly through changes in purchasing behavior and dietary patterns. Program awareness was included as a secondary process measure to assess initial reach and engagement, given the passive delivery model of the intervention. To ensure data integrity and reduce fraud or duplicate entries, a multilayered verification strategy was used, which is detailed elsewhere.^20^

### Statistical Analysis

Summary statistics were computed by state and time period, with differences tested using Wald tests for continuous variables and χ^2^ tests for categorical variables. Linear regression difference-in-differences models were computed with robust standard errors clustered on participant. Models were computed with and without adjusting for participant characteristics. Minimally adjusted models adjusted for household receipt of WIC benefits in the past 3 months and participant employment. Fully adjusted models additionally adjusted for educational level, marital status, household size, living arrangement, race and ethnicity, gender identity, and baseline age. Stratified exploratory analyses examined participants above and below median baseline fruit and vegetable intake distribution (1.79 cup equivalents per 1000 kcal), with 363 participants with intake of 0.10 to 1.79 cup equivalents per 1000 kcal and 362 participants with intake of more than 1.79 to 7.13 cup equivalents per 1000 kcal. For all analyses, vegetable intake excludes legumes. Sensitivity analyses included participants with extreme dietary intakes who were excluded for the primary analyses. Analyses used Stata/MP, version 18.0 (StataCorp LLC). All *P* values were from 2-sided tests, and results were deemed statistically significant at *P* < .05.

## Results

The analytic sample included 725 SNAP participants (mean [SD] age, 34.5 [10.1] years). The cohort included 690 women (95.2%) and 35 men, nonbinary, or third-gender individuals (4.8%). Mean (SD) household size at baseline and follow-up was 3.9 (1.6). By race and ethnicity, participants were Hispanic (n = 289 [39.9%]), non-Hispanic Black or African American (n = 121 [16.7%]), non-Hispanic White (n = 243 [33.5%]), and non-Hispanic other race or ethnicity (n = 72 [9.9%]). Across Rhode Island and Connecticut, approximately half of participants were employed (Rhode Island at baseline, 153 of 364 [42.0%]; Connecticut at baseline, 178 of 361 [49.3%]) and approximately half had some college or more (Rhode Island, 170 of 364 [46.7%]; Connecticut, 217 of 361 [60.1%]). (Table 1). At baseline, participant characteristics were largely similar between Rhode Island and Connecticut, with the exception of small but statistically significant differences in the proportion receiving WIC benefits (Rhode Island, 279 of 364 [76.6%] vs Connecticut, 298 of 361 [82.5%]), employed (Rhode Island, 153 of 364 [42.0%] vs Connecticut, 178 of 361 [49.3%]), who completed some college or more (Rhode Island, 170 of 364 [46.7%] vs Connecticut, 217 of 361 [60.1%]), and differences in race and ethnicity. At follow-up, sample characteristics remained similar between states, apart from more Rhode Island participants reporting receipt of summer EBT for their children (221 of 364 [60.7%] vs 145 of 361 [40.2%]; *P* < .001). The differences observed at baseline for other variables were no longer statistically significant. The racial and ethnic composition of our sample was broadly like state-level SNAP populations in Rhode Island and Connecticut, with roughly comparable proportions of Hispanic and non-Hispanic White participants.^21^

**Table 1.** Characteristics of Analytical Sample of SNAP Participants in Rhode Island and Connecticut^a^

Characteristic	No. (%)				Difference, *P* value	
	Rhode Island (n = 364)		Connecticut (n = 361)			
	Baseline	Follow-up	Baseline	Follow-up	Baseline	Follow-up
Participant and household characteristics						
Gender identity						
Women	342 (94.0)	NA	348 (96.4)	NA	.13	NA
Men, nonbinary individuals, or third gender individuals	22 (6.0)		13 (3.6)			
Baseline age, mean (SD), y	35.2 (10.6)	NA	33.8 (9.5)	NA	.07	NA
Received WIC benefits in past 3 mo	279 (76.6)	245 (67.3)	298 (82.5)	254 (70.4)	.049	.38
Employed	153 (42.0)	171 (47.0)	178 (49.3)	190 (52.6)	.049	.13
Marital status						
Married or living with a partner	93 (25.6)	97 (26.7)	112 (31.0)	118 (32.7)	.22	.14
Never married, divorced, widowed, or separated	253 (69.5)	250 (68.7)	229 (63.4)	232 (64.3)		
Prefer not to answer	18 (5.0)	17 (4.7)	20 (5.5)	11 (3.1)		
Household size, mean (SD) No.	3.8 (1.6)	3.9 (1.6)	3.9 (1.6)	3.9 (1.6)	.37	.84
Living arrangement other than housing where own or pay to stay	36 (9.9)	24 (6.6)	38 (10.5)	32 (8.9)	.78	.25
Race and ethnicity						
Hispanic	144 (39.6)	NA	145 (40.2)	NA	<.001	NA
Non-Hispanic Black or African American	33 (9.1)		88 (24.4)			
Non-Hispanic White	145 (39.8)		98 (27.2)			
Non-Hispanic other^b^	42 (11.5)		30 (8.3)			
Completed some college or more	170 (46.7)	NA	217 (60.1)	NA	<.001	NA
Received summer EBT for children in past 3 mo						
Yes	NA	221 (60.7)	NA	145 (40.2)	NA	<.001
No		119 (32.7)		177 (49.0)		
Not sure		24 (6.6)		39 (10.8)		
Calorie-adjusted fruit and vegetable intake, mean (SD), cup equivalents/1000 kcal						
Total fruit (excluding juice) and vegetable (excluding legumes) intake	2.05 (1.19)	2.20 (1.31)	2.12 (1.25)	2.13 (1.26)	.43	.51
Total fruit intake excluding juice	0.97 (0.78)	1.05 (0.92)	0.99 (0.91)	1.00 (0.92)	.71	.49
Total vegetable intake excluding legumes	1.08 (0.70)	1.14 (0.73)	1.13 (0.70)	1.13 (0.67)	.36	.77
Healthy Eating Index–2015 score, mean (SD)	63.72 (11.77)	63.77 (11.61)	64.28 (10.94)	63.59 (11.26)	.51	.83

Abbreviations: EBT, Electronic Benefits Transfer; NA, not applicable; SNAP, Supplemental Nutrition Assistance Program; WIC, Supplemental Nutrition Program for Women, Infants, and Children.

^a^ There were 364 participants in Rhode Island and 361 participants in Connecticut for whom data was obtained on all relevant covariates at both time points and who were not excluded due to extreme reported dietary intakes (≤500 kcal, ≥5500 kcal, or ≤25 different foods from the food frequency questionnaire). Data on receipt of summer EBT for children were obtained only at follow-up because this program did not start in either Rhode Island or Connecticut until 2024, after baseline data collection was already complete. *P* values for the statistical significance of differences between Rhode Island and Connecticut at each time point were computed using Wald tests for the continuous variables and Pearson χ^2^ tests for the categorical variables.

^b^ Includes individuals who selected 1 of the following racial categories: American Indian or Alaska Native, Asian, Native Hawaiian, Other Pacific Islander, or do not know or not sure. It also includes individuals who selected more than 1 race or provided a self-described category that did not align with standard classifications (eg, Jewish, Italian, Cape Verdean, and Portuguese). Individuals who identified as Hispanic in the preceding ethnicity question were classified as Hispanic regardless of their response to the racial or ethnic background question.

For the difference-in-differences analyses, there were no meaningful changes in any sample characteristics in Rhode Island that were not mirrored by similar changes in Connecticut. The proportion of participants reporting recent WIC participation declined in both states from baseline to follow-up, from 76.6% (279 of 364) to 67.3% (245 of 364) in Rhode Island and from 82.5% (298 of 361) to 70.4% (254 of 361) in Connecticut, and the proportion who were employed increased slightly in both states, from 42.0% (153 of 364) to 47.0% (171 of 364) in Rhode Island and from 49.3% (178 of 361) to 52.6% (190 of 361) in Connecticut. Across both Rhode Island and Connecticut, more than 93% of participants identified as women (94.0% [342 of 364] in Rhode Island; 96.4% [348 of 361] in Connecticut). Household size was approximately 4 people at baseline (mean [SD], 3.8 [1.6] in Rhode Island and 3.9 [1.6] in Connecticut) and follow-up (mean [SD], 3.9 [1.6] in Rhode Island and 3.9 [1.6] in Connecticut). Approximately 40% of the participants identified as Hispanic (Rhode Island, 144 of 364 [39.6%]; Connecticut, 145 of 361 [40.2%]).

**Figure 2.**
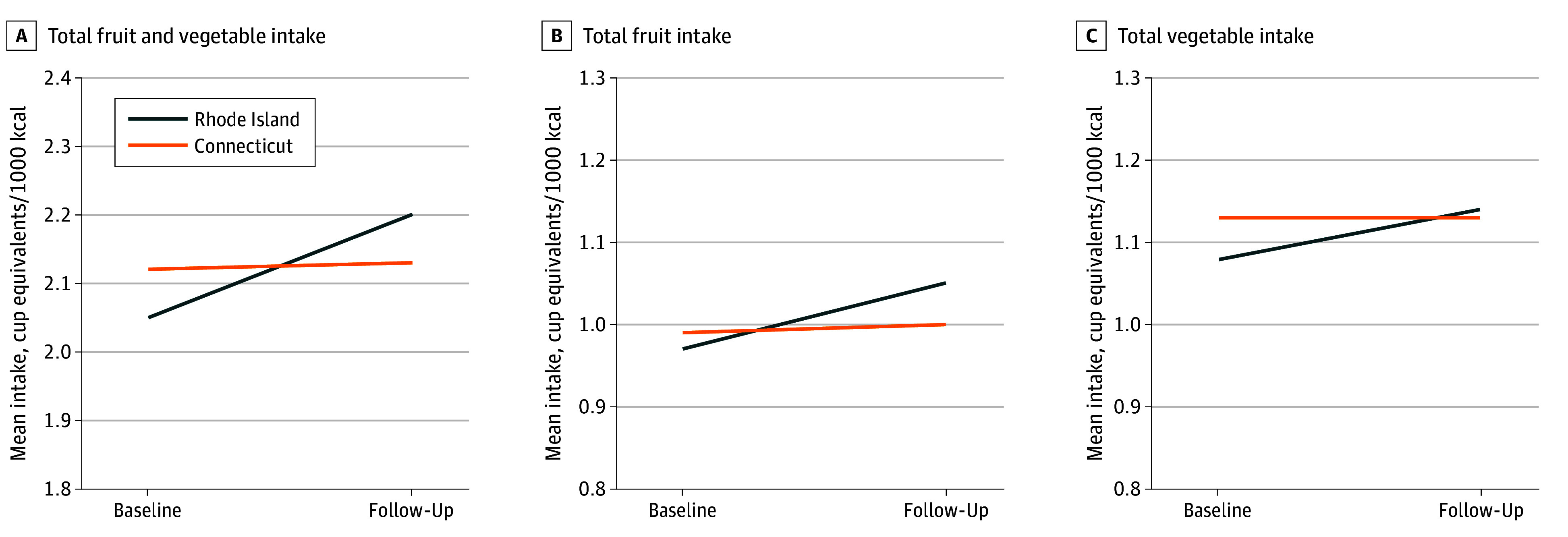
Calorie-Adjusted Fruit and Vegetable Intake Among Analytical Sample of Supplemental Nutrition Assistance Program Participants in Rhode Island and Connecticut Total fruit intake excludes juice and total vegetable intake excludes legumes.

Mean fruit and vegetable intake at baseline in both states was approximately 2.1 cup equivalents per 1000 kcal (mean [SD], 2.05 [1.19] in Rhode Island and 2.12 [1.25] in Connecticut) (Figure 2). In Rhode Island, calorie-adjusted fruit and vegetable intake increased from 2.05 to 2.20 cup equivalents per 1000 kcal, while Connecticut remained steady at 2.12 to 2.13 cup equivalents per 1000 kcal. The mean (SD) total Healthy Eating Index–2015 baseline scores were 63.7 (11.8) in Rhode Island and 64.3 (10.9) in Connecticut, which remained stable over time (mean [SD] follow-up scores, 63.8 [11.6] in Rhode Island and 63.6 [11.3] in Connecticut).

Difference-in-differences regression models comparing changes in dietary intake in Rhode Island relative to those in Connecticut showed statistically nonsignificant improvements in fruit and vegetable intake. In the fully adjusted model, Rhode Island participants’ consumption of total fruits and vegetables (excluding juice and legumes) increased by an estimated 0.12 (95% CI, −0.04 to 0.28) cup equivalents per 1000 kcal relative to changes among Connecticut participants (Table 2). This included a 0.06 (95% CI, −0.06 to 0.18) cup equivalent per 1000 kcal increase in fruit intake and a 0.06 (95% CI, −0.04 to 0.16) cup equivalent per 1000 kcal increase in vegetable intake. In stratified analyses, the changes seen with the Rhode Island intervention relative to changes in Connecticut differed significantly by baseline fruit and vegetable intake level. Among participants in the lower half of baseline intake (0.10-1.79 cup equivalents per 1000 kcal), there was no measurable change; Rhode Island participants’ consumption of total fruits and vegetables was stable (difference, 0.00 [95% CI, –0.17 to 0.18] cup equivalents per 1000 kcal) relative to changes among Connecticut participants (Table 3). In contrast, among those in the upper half of baseline intake, Rhode Island participants’ consumption of total fruits and vegetables increased (difference, 0.29 [95% CI 0.03-0.54] cup equivalents per 1000 kcal) relative to changes among Connecticut participants.

**Table 2.** Results of Difference-in-Differences Regression Models Examining Changes in Dietary Intake in Rhode Island Relative to Connecticut^a^

Outcome	Adjusted model, coefficient (95% CI)	
	Minimally	Fully
Total fruit (excluding juice) and vegetable intake, cup equivalents per 1000 kcal	0.13 (−0.03 to 0.29)	0.12 (−0.04 to 0.28)
Total fruit intake excluding juice, cup equivalents per 1000 kcal	0.07 (−0.06 to 0.19)	0.06 (−0.06 to 0.18)
Total vegetable intake, cup equivalents per 1000 kcal	0.06 (−0.04 to 0.17)	0.06 (−0.04 to 0.16)
Healthy Eating Index–2015 score	0.72 (−0.75 to 2.18)	0.68 (−0.80 to 2.16)

^a^ Difference-in-differences coefficients shown from linear regression models with robust standard errors clustered on participant. Models described in the Statistical Analysis section of the Methods.

**Table 3.** Results of Fully Adjusted Difference-in-Differences Regression Models Examining Changes in Dietary Intake in Rhode Island Relative to Connecticut, Stratified by Halves of Baseline Fruit and Vegetable Intake^a^

Outcome^b^	Coefficient (95% CI)
**Total fruit (excluding juice) and vegetable intake, cup equivalents per 1000 kcal**	
Baseline fruit and vegetable intake half 1 (0.10-1.79 cup equivalents/1000 kcal)	0.00 (−0.17 to 0.18)
Baseline fruit and vegetable intake half 2 (>1.79-7.13 cup equivalents/1000 kcal)	0.29 (0.03 to 0.54)^c^
**Total fruits (0-5)**	
Baseline fruit and vegetable intake half 1 (0.10-1.79 cup equivalents/1000 kcal)	0.04 (−0.34 to 0.43)
Baseline fruit and vegetable intake half 2 (>1.79-7.13 cup equivalents/1000 kcal)	0.01 (−0.22 to 0.25)
**Whole fruits (0-5)**	
Baseline fruit and vegetable intake half 1 (0.10-1.79 cup equivalents/1000 kcal)	0.16 (−0.20 to 0.53)
Baseline fruit and vegetable intake half 2 (>1.79-7.13 cup equivalents/1000 kcal)	0.00 (−0.17 to 0.17)
**Total vegetables (0-5)**	
Baseline fruit and vegetable intake half 1 (0.10-1.79 cup equivalents/1000 kcal)	0.03 (−0.26 to 0.32)
Baseline fruit and vegetable intake half 2 (>1.79-7.13 cup equivalents/1000 kcal)	0.05 (−0.15 to 0.24)
**Greens and beans (0-5)**	
Baseline fruit and vegetable intake half 1 (0.10-1.79 cup equivalents/1000 kcal)	0.02 (−0.35 to 0.39)
Baseline fruit and vegetable intake half 2 (>1.79-7.13 cup equivalents/1000 kcal)	0.36 (0.07 to 0.65)^c^

^a^ Difference-in-differences coefficients shown from linear regression models with robust standard errors clustered on participant. Models described in the Statistical Analysis section of the Methods.

^b^ Total fruit and vegetable intake is reported in cup equivalents per 1000 kcal. All other fruit and vegetable-related outcomes (total fruits, whole fruits, total vegetables, greens and beans) reflect Healthy Eating Index component scores ranging from 0 to 5 points. Higher scores are more concordant with dietary guidelines.

^c^ Statistically significant at *P* < .05.

The estimated change in total Healthy Eating Index–2015 score in Rhode Island relative to Connecticut was small and not statistically significant (0.68 [95% CI, −0.80 to 2.16]) (Table 2). Minimally adjusted models had slightly higher, but still nonsignificant, estimates. Sensitivity analyses including participants with extreme reported dietary intakes yielded similar results (eTable 1 in Supplement 1).

Program awareness was low, with 36.8% of respondents in Rhode Island (134 of 364) responding correctly to a question at follow-up asking about what the EWBW program does; another 36.8% (134 of 364) said they did not know, while 25.0% (91 of 364) said it teaches people about eating healthy foods, and 1.4% (5 of 364) said it gives kids free breakfast (eTable 2 in Supplement 1). When asked if they had used the discounts available through the EWBW, only 26.4% (96 of 364) said yes, while 28.8% (105 of 364) said no and 44.7% (163 of 364) said they did not know.

## Discussion

This study reports findings from a short-term evaluation of the EWBW SNAP fruit and vegetable incentive program. Despite incorporating best practices such as automatic enrollment and partnerships with major supermarkets, the program was not associated with statistically significant changes in fruit and vegetable intake. Results trended in the anticipated direction but were statistically significant only for participants with higher baseline fruit and vegetable consumption, underscoring heterogeneity in program responsiveness. Taken together, these null findings likely reflect unique characteristics of the sample as well as 3 major implementation factors: limited accessibility and reach, low participant awareness, and challenges with program integration.

Comparison with HIP helps contextualize our findings. HIP used a randomized clinical trial to test the effects of a 30% rebate on targeted SNAP-eligible fruit and vegetable purchases and found significant increases in intake of 0.18 cup equivalents/d at 4 to 6 months and 0.24 cup equivalents/d at 9 to 11 months.^8^ Despite offering a larger 50% incentive, EWBW was associated with a nonsignificant change in fruit and vegetable intake (0.12 cup equivalents/1000 kcal), suggesting that both program elements and implementation challenges may have contributed to null findings. Moreover, unlike HIP, our study population was heavily weighted toward households participating in the WIC program who already received monthly fruit and vegetable benefits through the WIC cash value voucher. Because WIC cash value voucher spending is administered separately from SNAP EBT and does not accrue EWBW rewards, these participants may have earned fewer incentives and shown smaller changes in fruit and vegetable intake. This compositional factor likely may have been associated with the overall estimated change and highlights the importance of accounting for overlapping program benefits when designing and evaluating SNAP-based incentives.

Regarding accessibility and reach, EWBW’s partnerships with only 2 grocery chains and allowing only in-store shopping may have limited participant options, particularly for households relying on discount grocers, corner stores, or dollar stores for food purchases. Although Stop & Shop represents 43% of market share in Rhode Island and 40% in Connecticut,^16^ it has also been shown to be more expensive than its competitors, which may affect SNAP households more than households with higher incomes. Walmart, on the other hand, is more affordable, but only has 2 outlets in Rhode Island that carry fresh produce. At the same time, recent research highlights the importance of dollar stores in food purchasing patterns, including fruits and vegetables, among households with low incomes,^22^ suggesting that broader retail partnerships could significantly improve program reach.

The awareness gap represents another important implementation challenge. Only approximately one-third of EWBW participants were able to identify the program’s purpose, compared with 62% to 76% awareness rates in HIP.^10^ This finding suggests that many eligible households either did not know about the program or did not understand how it worked. As behavior change frameworks emphasize, knowledge and understanding are prerequisites for use, and financial incentives alone are unlikely to succeed without effective communication and engagement strategies.^23^

Finally, program integration may have played a role. The EWBW program implemented an evidence-based structural nudge through automatic enrollment, which reduced barriers to entry. However, consistent with behavioral economics research, defaults are a necessary but not sufficient condition for behavior change.^14,15^ Participants navigating complex food assistance systems may require multiple layers of support, including clear point-of-sale prompts, reinforcement across retail environments, and seamless integration with existing shopping routines. The program’s restriction to fresh produce only may have further limited integration into participants’ shopping habits, particularly for households with constrained capacity for food storage or limited cooking facilities. Without such features, automatic enrollment alone may be insufficient to drive meaningful changes in behavior.

Our finding that participants with higher baseline fruit and vegetable intake but not those in the bottom half of the distribution experienced overall increases in fruit and vegetable intake suggests that individuals who are already motivated to eat healthfully are particularly responsive to financial incentives. Several factors may help explain this pattern. First, these individuals likely have established preferences and habits around consuming fruit and vegetables, making it easier to increase intake when affordability improves. They may also possess greater food literacy and cooking skills, enabling them to incorporate additional produce into meals with minimal effort.^24^ They may also perceive lower risk in purchasing perishable items and be less concerned about food waste.^25^ Future research should aim to better understand how these factors shape differential responsiveness to incentive programs and whether targeted strategies can help individuals with lower intake of fruits and vegetables benefit to the same extent.

### Limitations

Several limitations of this study should be acknowledged. The 5- to 8-month period may have insufficiently captured maximum program outcomes due to lower initial awareness and other store-level factors. The FFQ has not been specifically validated among populations with lower incomes and, while it assessed usual intake over the past 3 months, it may not have been sensitive enough to detect smaller changes in fruit and vegetable intake. Data collection was self-administered and many participants required follow-up by study staff to complete or clarify responses; despite extensive quality assurance procedures, it is possible that some errors or incomplete responses were missed. Although having multiple preintervention data points would have enabled a more rigorous assessment of the parallel trends assumption, similarities in the demographic and socioeconomic characteristics across the Northeast region, coupled with the absence of other major policy or environmental changes during the study period, support the expectation that observed relative changes are associated with the intervention. The study was also slightly underpowered relative to the original target. The final analytic sample included 361 to 364 participants per state, which, under the same assumptions, provided power to detect a 0.27-cup difference in fruit and vegetable intake (close to the 0.25-cup difference specified a priori). Finally, the generalizability of these findings is limited by several factors, including our convenience sample which was heavily weighted toward WIC-participating households.

## Conclusions

This cohort study found no overall short-term significant increase in fruit and vegetable intake after the implementation of the EWBW program. However, stratified analyses revealed that participants with higher baseline intake of fruits and vegetables experienced a significant improvement, suggesting that financial incentives may be most effective among individuals already inclined toward healthy eating. Future research should examine the implementation processes and contextual factors that may have limited broader impact, such as low program awareness, barriers to participation, or varying levels of community engagement. Longer-term follow-up is also needed to assess sustained changes in dietary behaviors and to explore potential spillover effects on other household members, particularly children, who may benefit indirectly from increased household access to fruits and vegetables. This study contributes to a growing body of evidence assessing fruit and vegetable incentive programs.^14^ With increased federal investment through programs like the GusNIP, such interventions, including Double Up Food Bucks and produce prescription programs, are being deployed across diverse clinical and community settings. These developments highlight the need for continued innovation and rigorous evaluation to identify best practices that can maximize reach and effectiveness.

## Notes

Supplement 1. **eTable 1.** Results of Difference-in-Differences Regression Models Examining Changes in Dietary Intake in Rhode Island Relative to Connecticut, Full Sample Without Exclusions for Extreme Dietary Intake **eTable 2.** Awareness and Use of the Eat Well, Be Well (EWBW) Incentive Program at Follow-Up Among Rhode Island Supplemental Nutrition Assistance Program (SNAP) Participants

Supplement 2.
Data Sharing Statement
