# Supplementary material for: Evaluation of a State-Level Incentive Program to Improve Diet
Source: JAMA Netw Open. 2025 Nov 18;8(11):e2544215. doi: 10.1001/jamanetworkopen.2025.44215 (PMC12628101; doi:10.1001/jamanetworkopen.2025.44215)
Supplement: Supplement 1. — eTable 1. Results of Difference-in-Differences Regression Models Examining Changes in Dietary Intake in Rhode Island Relative to Connecticut, Full Sample Without Exclusions for Extreme Dietary Intake eTable 2. Awareness and Use of the Eat Well, Be Well (EWBW) Incentive Program at Follow-Up Among Rhode Island Supplemental Nutrition Assistance Program (SNAP) Participants [file jamanetwopen-e2544215-s001.pdf]

Supplementary Online Content

Tovar A, Powell LM, Leider J, Elenio E, Oddo VM, Vadiveloo MK. Evaluation of a state-level incentive program to improve diet. *JAMA Netw Open.* 2025;8(11):e2544215. doi:10.1001/jamanetworkopen.2025.44215

eTable 1. Results of Difference-in-Differences Regression Models Examining Changes in Dietary Intake in Rhode Island Relative to Connecticut, Full Sample Without Exclusions for Extreme Dietary Intake

eTable 2. Awareness and Use of the Eat Well, Be Well (EWBW) Incentive Program at Follow-Up Among Rhode Island Supplemental Nutrition Assistance Program (SNAP) Participants

This supplementary material has been provided by the authors to give readers additional information about their work.

© 2025 Tovar A et al. *JAMA Network Open.*

eTable 1. Results of Difference-in-Differences Regression Models Examining Changes in Dietary Intake in Rhode Island Relative to Connecticut, Full Sample Without Exclusions for Extreme Dietary Intake

	Coefficient (95% CI)
Total fruit (excluding juice) and vegetable intake, cup equivalents per 1000 kcal	0.10 (-0.08-0.27)
Total fruit intake excluding juice, cup equivalents per 1000 kcal	0.06 (-0.06-0.18)
Total vegetable intake, cup equivalents per 1000 kcal	0.03 (-0.09-0.15)
Healthy Eating Index (HEI)-2015 Score	0.45 (-0.94-1.85)

N=833 Rhode Island and Connecticut SNAP participants. Difference-in-differences coefficients shown from linear regression models with robust standard errors clustered on participant adjusting for household receipt of WIC in the past 3 months, education, employment, marital status, household size, living arrangement, race and ethnicity, gender, and baseline age. Vegetable intake excludes legumes.

© 2025 Tovar A et al. *JAMA Network Open.*

eTable 2. Awareness and Use of the Eat Well, Be Well (EWBW) Incentive Program at Follow-Up Among Rhode Island Supplemental Nutrition Assistance Program (SNAP) Participants[a]

	n (%)
What does EWBW do?	
Gives adults in Rhode Island who use food stamps/SNAP benefits a discount to buy fresh fruits and vegetables[b]	134 (36.8)
Teaches people about eating healthy foods	91 (25.0)
Gives kids free breakfast	5 (1.4)
Don't know	134 (36.8)
Used discounts available through EWBW?	
Yes	96 (26.4)
No	105 (28.9)
Don't know	163 (44.8)

[a]N=364 Rhode Island SNAP participants.

[b]This was the correct response.

© 2025 Tovar A et al. *JAMA Network Open.*
