# Supplementary material for: Evaluation of a State-Level Incentive Program to Improve Diet
Source: JAMA Netw Open. 2025 Nov 18;8(11):e2544215. doi: 10.1001/jamanetworkopen.2025.44215 (PMC12628101; doi:10.1001/jamanetworkopen.2025.44215)
Supplement: Supplement 2. — Data Sharing Statement [file jamanetwopen-e2544215-s002.pdf]

Data Sharing Statement

Tovar. Evaluation of a State-Level Incentive Program to Improve Diet. *JAMA Netw Open*. Published November 18, 2025. doi:10.1001/jamanetworkopen.2025.44215

Data
Data available: No

Additional Information
Explanation for why data not available: Per our IRB stated that data can not be shared.
